# Foliar spray of salicylic acid and gamma-aminobutyric acid mitigates chromium toxicity in barley by regulating antioxidant defense, nutrient uptake, and secondary metabolites

**DOI:** 10.1186/s12870-026-09105-5

**Published:** 2026-06-13

**Authors:** Leeza Mujahid, Muhammad Umer Chattha, Muhammad Naeem Sattar, Imran Khan, Muhammad Bilal Chattha, Adeeba Zafar, Abdul Ghafoor, Hesham S. Ghazzawy, Basem Abdelraheem Rasheed Ayoub, Sallah A. Al Hashedi

**Affiliations:** 1 Department of Botany, University of Agriculture, Faisalabad, 38040 Pakistan; 2 Department of Agronomy, University of Agriculture, Faisalabad, 38040 Pakistan; 3 Central Laboratories, King Faisal University, P.O. Box 420, Al-Ahsa, 31982 Saudi Arabia; 4 Department of Agronomy, University of the Punjab, Lahore, 54590 Pakistan; 5 Center for Water and Environmental Studies, King Faisal University, Al- Ahsa, 31982 Saudi Arabia; 6 Date Palm Research Center of Excellence, King Faisal University, Al-Ahsa, 31982 Saudi Arabia; 7 Institute of Studies and Consultations, King Faisal University, Al-Ahsa, 31982 Saudi Arabia

**Keywords:** Antioxidants, Chromium toxicity, Oxidative stress, Barley, Yield

## Abstract

**Background:**

Chromium (Cr) contamination severely hinders plant growth and biomass production. Salicylic acid (SA) and gamma-aminobutyric acid (GABA) play a vital role in improving plant stress resilience. So for, their synergistic effects in mitigating Cr toxicity remain unexplored. Thus, this research aimed to examine the effects of SA and GABA in mitigating the toxic effects of Cr in barley.

**Methodology:**

The study included Cr stress (0 and 40 mg kg^− 1^), two barley cultivars (Sultan-17 and Haider-93), and foliar sprays of SA (1 mM) and GABA (1 mM).

**Results:**

Chromium toxicity significantly decreased growth and yield in both cultivars by increasing hydrogen peroxide (H_2_O_2_: 84%), Cr accumulation, and decreasing leaf water content (37–42%), chlorophyll synthesis (36–40%), and nutrient uptake. The foliar spray of SA + GABA markedly retrieved the toxic impacts of Cr by minimizing the Cr accumulation in roots (54–58%), leaves (46–50%), H_2_O_2_ (55–62%), increasing the chlorophyll synthesis, leaf water contents (52–59%), osmolytes synthesis, and antioxidants activities (9–91%), thereby improving the growth and yield. Additionally, SA + GABA improved the secondary metabolites production and maintained the ionic balance by increasing the nutrient uptake, thereby improving the barley growth and yield. Overall, cultivar Sultan-17 performed appreciably well compared to Haider-93 owing to its better potential to counteract Cr.

**Conclusion:**

Thus, co-applied SA and GABA mitigate Cr toxicity by improving antioxidant defense, photosynthetic pigments, secondary metabolites production, and restricting the Cr uptake. These findings provides foundation to develop eco-friendly and economical measures to enhance crop productivity and contaminated soils.

## Introduction

Heavy metals (HM) pollution is a serious concern globally owing to their long-term persistent and ability to degrade soil quality, crop productivity, and human health [1–3]. Different anthropogenic actions, including industrialization, mining, and agricultural practices has increased the accumulation of HM in soils, which are negatively affecting crop productivity and the food chain [4, 5]. Among different HM, chromium (Cr) is one of the most abundant hazardous metals existing in agricultural lands with no vital role in plant metabolism [5]. Chromium is the second most abundant metal found in soils, negatively affecting soils and groundwater, posing serious health risks [6]. The mining activities and industrial effluents are major sources of Cr discharge into the soil environment; therefore, it diminish the quality and productivity of major agricultural crops [7]. Chromium has different oxidation states, and it usually exists in Cr^3+^ and Cr^6+^ forms. Among these, Cr^3+^ is considered as less hazardous and at lower concentrations it is function as an essential trace element for the metabolism of glucose and lipid in living organisms [8]. Conversely, Cr^6+^ exhibits high toxicity, and it is absorbed by plants via membrane sulfate transport channels, where it induces extensive effect to lipids, DNA, and proteins via the production of reactive oxygen species (ROS), histone modification, and methylation [5, 9]. Chromium also delays seed germination, reduces root growth, biomass, damage photosynthetic apparatus and membranes and eventually cause plant death [10]. It also affects the growth and development of floral organs and fruits, thus compromising the crop quality [11]. Moreover, it also impairs the water and nutrient uptake, thereby diminishing the plant growth and yield [12]. However, plants use different mechanisms such as antioxidants and Cr sequestration to reduce its damaging impacts [13]. The effectiveness of these mechanisms varies across different plant species [14].

Globally, diverse strategies are used to counteract HM toxicity. Among these strategies, exogenous supplementation of signaling molecules and pyto-hormones have emerged as a rising star to counteract HM toxicity. Salicylic acid (SA) is a phenolic substance present throughout the plant kingdom. It functions as a modulator of numerous biochemical and physiological activities, including thermogenesis, signaling, and plant growth, defense mechanism and responses to stress conditions [15]. Salicylic acids also regulates antioxidants activities [16] and suppresses lethal effect of HM by modulating their translocation and absorption [17]. It also scavenges the ROS by regulating the antioxidant enzyme activities, thereby preserve chlorophyll content and improve photosynthetic efficiency [18]. Moreover, it also supports osmotic adjustment by increasing proline and other compatible solutes accumulation, therefore, stabilizes cellular membranes, and enhance resilience to HM stress [18].

Gamma-aminobutyric acid (GABA), is an important acid playing a crucial role in developmental and growth processes in plants under both stressful and normal conditions [19]. The biosynthesis of GABA has been documented at various developmental stages, particularly during vegetative and reproductive phases and in response to stress exposure [20]. It strengthens antioxidant defense and the glyoxalase system, hence contributing to MG detoxification [21]. It stimulates development and stress tolerance through boosting antioxidant machinery; thus, resulting in improving crop performance [22]. Application of GABA enhances internal GABA level and activates multiple levels of molecular, physiological, and biochemical responses [23]. Moreover, it regulates the levels of nicotinamide synthase, metallothionein, and amino acids, which form stable complexes with heavy metals, thus controlling their uptake of heavy [24].

Barley (*Hordeum vulgare* L.) is a major grain crop of the Poaceae family, used for animal feed and human consumption [25]. Barley is identified by its health promoting effects in combating degenerative disorders, consisting of obesity, sugar, colon inflammation, and high blood pressure [26]. Although individual roles of SA and GABA in mitigating Cr stress are well studied, their synergistic effects in mitigating Cr toxicity are unexplored. No study has been conducted to determine how combining SA and GABA application alleviates Cr toxicity. This study fills these gaps by showing that co-applied SA and GABA synergistically decrease Cr uptake and upregulate antioxidant defense, nutrient uptake, and secondary metabolites under Cr stress. We hypothesized that combined SA and GABA treatment would synergistically mitigate Cr toxicity in barley plants by limiting Cr uptake and translocation, boosting antioxidant defense, physiological and biochemical functioning, and nutrient homeostasis. The objectives of the current study were as follows: i) to determine the impact of SA and GABA on barley growth and yield, 2) to assess the impacts of SA and GABA on plant physiological and biochemical functioning and nutrient homeostasis, 3) to determine the effect of SA and GABA in reducing the Cr accumulation in barley plants. This study provides insights to develop eco-friendly and cost-effective measures to mitigate Cr toxicity and improve crop productivity in contaminated soils. 

## Materials and methods

### Experiment site and treatments

A pot experiment was conducted in the wire house of University of Agriculture, Faisalabad during the Rabi season 2024–2025. The experiment site has a semi-arid climate with hot summers and cold winters. The study site has a mean maximum temperature of 28 °C and a mean minimum temperature of 25 °C during the growing period. The study comprised Cr stress (0 and 40 mg kg^− 1^ soil), foliar spray of SA (1 mM) and GABA (1 mM), and two barley cultivars: Sultan-17 and Haider-93. The seeds of Sultan-17 and Haider-93 were obtained from Ayub Agricultural Research Institute Faisalabad, Pakistan. The experiment was arranged in a completely randomized design (CRD) with factorial arrangement containing three replications. Soil was taken from Agronomy field (0–20 cm), and the soil was mixed with silt in 2:1 ratio. The soil was alkaline with a pH of 7.44, organic matter content of 0.91%, total nitrogen content of 0.044%, available phosphorous and available potassium contents of 13.11 and 165.11 mg kg^− 1^, respectively. The plastic pots (length: 46 cm, diameter: 25 cm) were filled with 8 kg dry soil. Chromium salt (K_2_Cr_2_O_7_) was mixed in soil according to the respective treatments. Thereafter, a field capacity level of 70% was maintained in pots, and they were placed in dark conditions for 8 weeks. Then, 12 seeds of barley were sown in every pot, and after germination, only five seedlings were retained in every pot. The recommended practices were adapted during the growing season to get healthy stand establishment. Salicylic acid and Gamma-aminobutyric acid were applied as foliar spray after 45 days of sowing, two times at three-day intervals.

### Measurement of morphological attributes

Plant height was recorded using a meter rod from three randomly selected plants per pot, and the average was calculated. Similarly, the identical three plants were uprooted cautiously, and roots and shoots were separated. Then, for the removal of soil, roots were cleaned thoroughly with distilled water, and after that, the length was measured and averaged. Also, the shoots’ length was measured using a meter rod, and the average was calculated. Moreover, to determine their fresh weight, the shoots and roots were weighed and followed by drying (70 °C) until constant weight to obtain dry weight.

### Determination of physiological and photosynthetic traits

One g of fresh leaves was measured on an electric balance (FW) and soaked in distilled water for 24 h. Then, again the weight of leaves was measured to calculate the turgid weight (TW). To achieve dry weight, the same leaves were dried using an oven (70 °C) for 24 h, and the RWC was estimated based on the method reported by [27] with the following formula:

$$\mathrm{RWC}\%=\left(\mathrm{FW-DW}\right)/\left(\mathrm{TW-DW}\right)\times100$$

Electrolyte leakage was measured using the procedure of Chattha et al. [28]. A freshly cut leave sample were cut into little pieces and soaked for 30 min in distilled water, and later EC1 was measured. Moreover, in a water bath, the samples were exposed to heat treatment at 90 °C for 50 min to measure the EC2, and the electrolyte leakage (%) was quantified by using the formula:

$$\mathrm{EC}\%=\left(\mathrm{EC}_{1}\right)/\left(\mathrm{EC}_{2}\right)\times100$$

A sample of 0.5 g of fresh leaves were taken and ground by using a pestle and mortar in 80% methanol solution. The extract was then centrifuged and filtered, and the absorbance was measured using a spectrophotometer at 663, 645, and 480 nm wavelengths, which were recorded for chlorophyll a, b, and carotenoids, respectively [29].

### Measurements of antioxidants, stress markers, osmolytes and secondary metabolites

For peroxidase (POD), catalase (CAT), superoxide dismutase (SOD), and ascorbate peroxidase (APX) activity, a sample of 0.5 g leaves was subjected to homogenization in 5 ml of potassium phosphate buffer. For the determination of CAT activity Aebi's [30], approach was used. To do this 0.1 mL of supernatant, 0.1 mL of H_2_O_2_, and 2.5 mL buffer were mixed, and on a spectrophotometer, absorbance was measured at 240 nm. For the assay of POD activity, 100 µL of enzyme extract was added to a reaction mixture, and the change in absorbance at 470 nm was recorded [31]. APX activity was determined by using the protocol of [32] after preparing enzymes mixture and reading the absorbance at 290 nm. The activity of SOD was measured with the methods of [33] method, based on inhibition of NBT photo-reduction. The hydrogen peroxide (H₂O_2_) was determined by [34]. Leaf sample of 0.5 g was blended in 1 mL of 0.1% trichloroacetic acid (TCA) and centrifuged at 10,000 rpm for 15 min at 4 °C.

Supernatant was transferred in Eppendorf tube and mixed with 0.1 mL of 10 M potassium phosphate buffer (pH 7.0), and absorbance was read at 390 nm. For MDA 0.5, barley leaves were homogenized by using the 5 mL trichloroacetic acid (TCA), then they were centrifuged (12,000) at 4 °C. Later, standard methods of [35] were followed, and reading measured at 532 and 600 nm to measured MDA contents. The leaf samples were ground in PPB and centrifuged (14,000) for 15 min. Thereafter, the extract was taken and mixed with 2 mL Bradford solution, and the reading was taken at 595 nm [36]. To measure, FA 1 mL extract was taken and added with 1 mL of pyridine and 1 mL of ninhydrin. The samples were transferred into a water bath (90 °C), and after 30 min, the samples were taken out. Later, they were mixed with water and reading measured at 570 nm [37]. The concentration of leaf phenolics in barley plants was measured with the methods of [38] after reading absorbance at 740 nm. For flavonoids, leaves were taken and homogenized. The mixture was taken, which was mixed with 0.1 M sodium nitrite (5.5 pH), and then it was subjected to heating for 10 min. After the heaving mixture was added to aluminum chloride (28 µL) and left for 10 min. Then it was mixed with 1 mM of NaOH (120 µL), and later reading was done at 510 nm [39, 40]. The leaves of barley plants were taken and cut into pieces. Then the leaves were added to 5% HClO_4_ (20 mL) solution and placed at -70 °C. Later concentration of putrescine (Put), spermidine (Spd), and spermidine (Spd) was estimated following the procedures of [41] after making some minor changes as indicated by [42].

### Yield attributes

Yield attributes such as tillers and spike length were manually collected and averaged. Moreover, to measure the biological yield, harvested plants were weighed and after that, threshed spikes from per plant were used to record the grains yield. A sub-sample of grains was collected and weighed to measure the grain weight. 

### Determination of chromium concentration in roots, leaves and grains

Oven-dried samples (roots, leaves, and grains) were then grind to a fine powder and digested using a 2:1 ratio of HNO_3_ and HClO_4_, as explained by [43]. The level of Cr in samples was measured using atomic absorption spectrophotometry (AAS. PerkinElmer Analyst™ 800). The following formula was used to find out the content of chromium in different plant parts (ug g^−^¹ of dry matter): Cr concentration = (AAS reading) (dilution factor)/ dry weight of sample. The concentration of N, P, and in digested samples was estimated with the Kjeldahl and spectrophotometer methods, and Ca, K, and Mg was measured with a flame photometer. 

### Statistical analysis

The recorded data were analyzed using the analysis of variance (ANOVA) technique implemented in STATISTIX 8.1. Treatment means were compared for significance at the 5% probability level using the honestly significant difference (HSD) test [44]. The heat-map was generated by using the pheatmap package in R-studio. For this, the data were normalized with Z-score transformation, and clustering was performed by using the Euclidean distance. The PCA was performed by using R, and the first two principal components were retained for visualization. 

## Results 

### Growth attributes

Chromium toxicity negatively affected the morphological attributes of barley cultivars (Table 1). The minimum shoot and root length was observed in Haider-93, and the maximum was observed in Sultan-17 under Cr toxicity. Salicylic acid + GABA alleviated the toxic impacts of Cr. This treatment combination increased shoot and root length by 40% and 58% in Sultan-17 and 51% and 59% in Haider-93, respectively, relative to the control (Table 1). Co-foliar spray of SA and GABA showed 49% enhancement in SFW, 45% enhancement in RFW, 63% enhancement in SDW and 77% enhancement in RDW in Sultan-17 and whereas this combination increased aforementioned traits by 55%, 46%, 83% and 79% in Haider-93 compared to control (Table 1).

**Table 1:** Effect of exogenous application of salicylic acid (SA) and gamma aminobutyric acid (GABA) on morphological parameters of two barley cultivars grown under chromium stress conditions

Varieties	Treatments	PH (cm)	SL (cm)	RL (cm)	SFW(g)	RFW(g)	SDW(g)	RDW(g)
Sultan-17	Control	69.50 ± 0.364a	56.48 ± 0.262a	3.56 ± 0.153a	28.89 ± 0.33a	1.65 ± 0.081a	7.96 ± 0.018a	0.97 ± 0.047a
	Cr	38.52 ± 0.581 g	35.91 ± 0.301f	2.13 ± 0.023 h	17.36 ± 0.16 h	1.02 ± 0.021 h	3.87 ± 0.247 h	0.48 ± 0.032 h
	Cr + SA	49.83 ± 0.081e	42.01 ± 0.310d	2.79 ± 0.312f	21.57 ± 0.23f	1.15 ± 0.102f	4.88 ± 0.042f	0.61 ± 0.042f
	Cr + GABA	58.64 ± 0.569c	46.33 ± 0.022c	3.13 ± 0.410d	23.74 ± 0.08d	1.32 ± 0.223d	5.63 ± 0.034e	0.72 ± 0.021d
	Cr + SA + GABA	64.82 ± 0.029b	50.30 ± 0.250b	3.66 ± 0.021b	26.01 ± 0.26b	1.48 ± 0.311b	6.33 ± 0.012c	0.85 ± 0.027b
Haider-93	Control	69.45 ± 0.329a	55.26 ± 0.640a	3.51 ± 0.167a	28.72 ± 0.37a	1.63 ± 0.267a	7.65 ± 0.155b	0.96 ± 0.098a
	Cr	34.43 ± 0.770 h	31.42 ± 0.287 g	2.02 ± 0.342i	16.36 ± 0.05i	0.96 ± 0.056i	3.24 ± 0.012i	0.44 ± 0.044i
	Cr + SA	46.13 ± 0.078f	37.78 ± 0.491e	2.69 ± 0.421 g	20.97 ± 0.20 g	1.10 ± 0.245 g	4.25 ± 0.015 g	0.58 ± 0.032 g
	Cr + GABA	55.32 ± 0.587d	43.09 ± 0.440d	3.00 ± 0.025e	22.85 ± 0.32e	1.20 ± 0.098e	5.13 ± 0.021f	0.68 ± 0.042c
	Cr + SA + GABA	61.28 ± 0.081c	47.73 ± 0.132c	3.22 ± 0.315c	25.39 ± 0.02c	1.41 ± 0.247c	5.95 ± 0.034d	0.79 ± 0.021c

Values represent means of three replicates ± SE

*C*_r_ 40 mg kg^− 1^ soil, *SA* 1 mM, *GABA* 1 mM, *PH* Plant height, *SL* Shoot length, *RL* Root length, *SFW* Shoot fresh weight, *RFW* Root fresh weight, *SDW* Shoot dry weight, *RDW* Root dry weight

Means followed by different letters are significantly different at *p* < 0.05

### Photosynthetic pigments

**Fig. 1:**
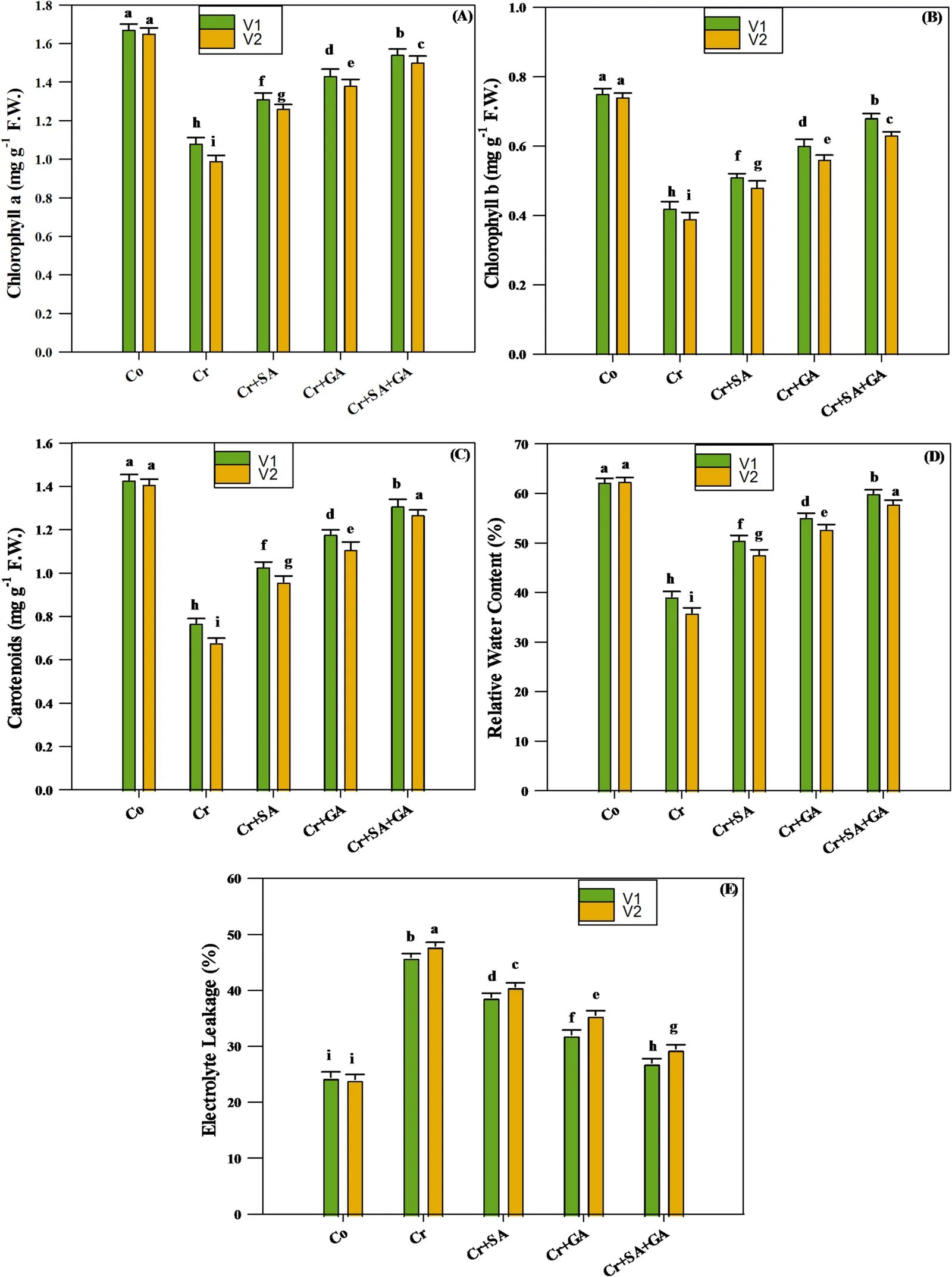
Effect of salicylic acid and GABA on chlorophyll (**a** and **b**), carotenoids (**c**), leaf water contents (**d**) and electrolyte leakage (**e**) of barley plants subjected to stress (40 mg kg^− 1^ soil). Each vertical bar represents the means of three replications ± SE. Bars bearing different letters differ significantly at *p* < 0.05. Chl: chlorophyll. V1: Sultan-17, V2: Haider-93

The chlorophyll a and chlorophyll b content reduced by 36% and 40% in sultan-17 and Haider-93 under Cr stress compared with control (Fig. 1). The carotenoids content also decreased by 47% and 52% in sultan-17 and Haider-93 in comparison to the control under Cr stress (Fig. 1). Foliar spray of SA and GABA significantly enhanced the chlorophyll a content, chlorophyll b, and carotenoid content in Sultan-17 and Haider-93, respectively, relative to their control (Fig. 1).

### Relative water content and electrolyte leakage

Chromium toxicity reduced RWC by 37% and 42% in Sultan-17 and Haider-93 under Cr stress. Conversely, under Cr stress, electrolyte leakage showed an increase of 87% and 98% in Sultan-17 and Haider-93, respectively, relative to control (Fig. 1). Co-foliar spray of SA + GABA improved RWC by 52% and 59%, and decreased the EL by 42% and 61% in Sultan-17 and Haider-93 under Cr stress than control (Fig. 1). 

### Antioxidant activities, osmolytes, oxidative markers and secondary metabolites

**Fig. 2:**
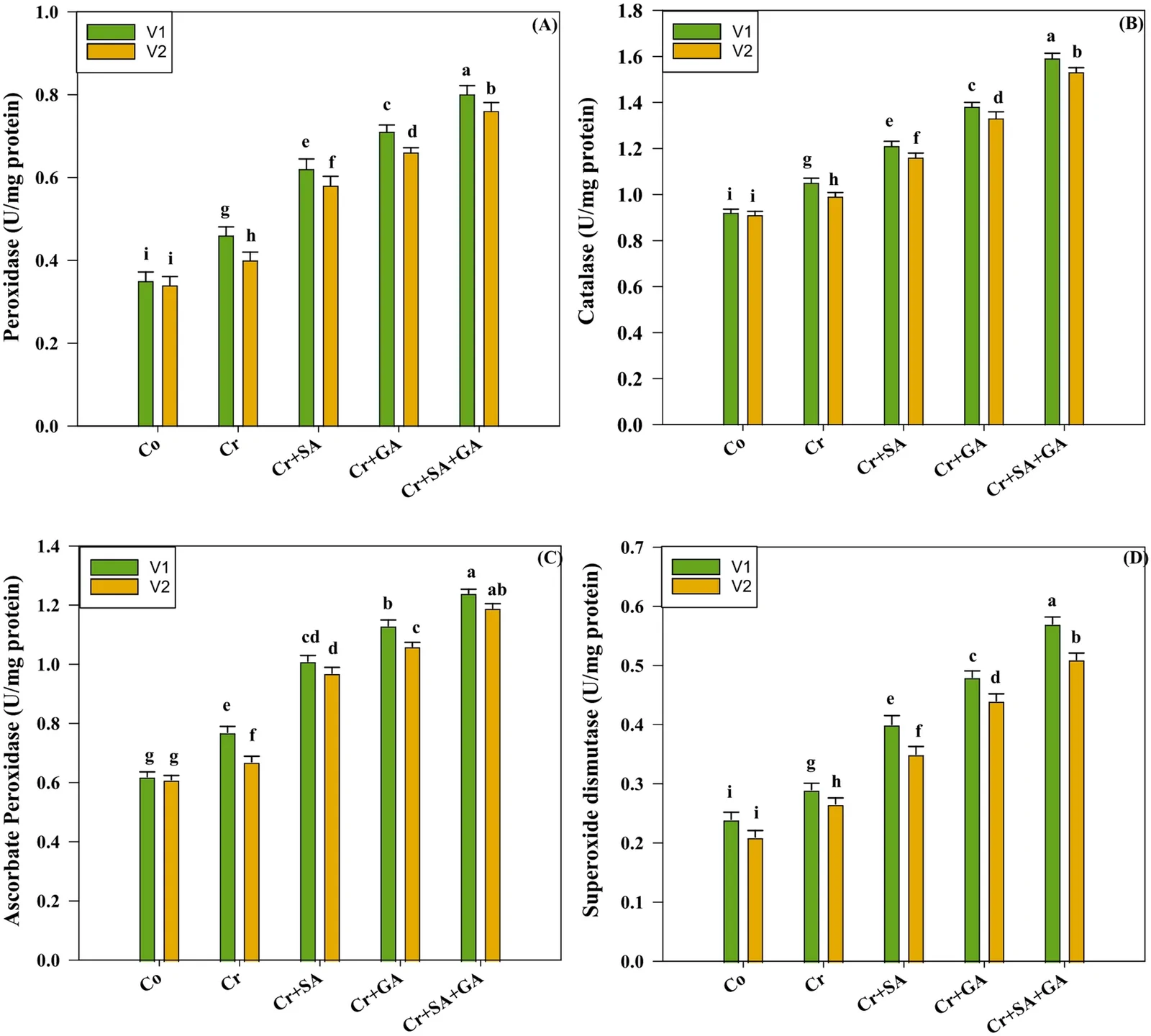
Effect of salicylic acid and GABA on the activity of (**a**) POD, (**b**) CAT, (**c**) APX and (**d**) SOD under Cr stress (40 mg/kg soil) stress. Error bars show the mean value of three replications ± S.E and different values shows significant differences (*P* ≤ 0.05) according to HSD test. V1: Sultan-17, V2: Haider-93

Chromium stress increased antioxidant activities, which were further increased with co-foliar application of SA and (Fig. 2). CAT activity increased by 14% and 9%, where POD activity increased by 31% and 16% in Sultan-17 and Haider-93, respectively, with respect to control (Fig. 2). Combined SA and GABA enhanced the CAT activity by 51% and 54%, and POD activity by 72% and 91% under Cr stress, compared with the control plant (Fig. 2). The Cr also increased the APX activity by 24% and 9% in Sultan-17 and Haider-93, respectively, with respect to control (Fig. 2). Under Cr stress, combined foliar supplementation enhanced the APX activity by 61% and 77% in Sultan-17 and Haider-93, respectively, relative to control (Fig. 2). Similarly, SOD content was increased in Cr stress, which was further increased owing to the combined exogenous application of SA+GABA (Fig. 2).

**Fig. 3:**
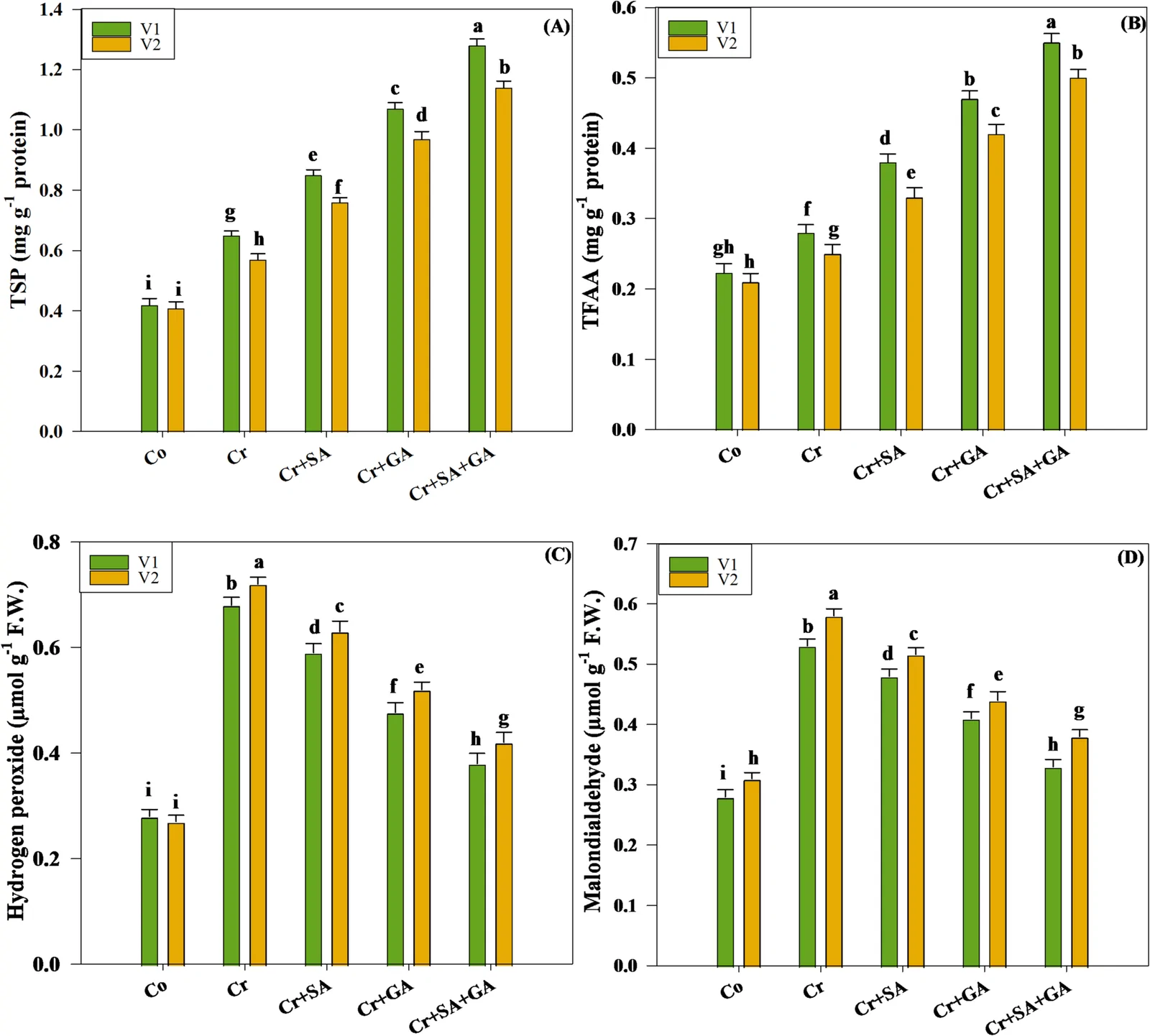
Impact of salicylic acid and GABA on osmolytes (**a** and **b**) and stress markers (**c** and **d**) of barley plants subjected to Cr stress (40 mg/kg soil). Vertical bars represent mean values ± SE, and columns annotated with different letters indicate statistically significant difference at *p* < 0.05. V1: Sultan-17, V2: Haider-93

TSP was reduced by 47% and 60% in Sultan-17 and Haider-93 under Cr stress in comparison to the control (Fig. 3). SA + GABA enhanced TSP by 97% and 98% in sultan-17 and Haider-93 under Cr stress as compared to control (Fig. 3). Likewise, FA also reduced by 25% and 17% in Sultan-17 and Haider-93 under Cr stress as compared to the control (Fig. 3). Salicylic acid + GABA increased the FAA by 96% and 98% in Sultan-17 and Haider-93 respectively, with respect to control (Fig. 3).

The concentration of H_2_O_2_ and MDA content increased by 84% in Sultan-17 and 89% in Haider-93 under Cr stress compared to control (Fig. 3). The combined spray of SA and GABA decreased the H_2_O_2_ content by 55%, 62% in Sultan-17, and 58%, 65% in Haider-93 under Cr stress relative to control (Fig. 3). The presented results displayed that synthesis of phenolic, flavonoids, putrescinc, and spermidine was significantly increase in cultivars Sultan-17 and Haider-93 in response to Cr stress compared to control (Table 2). Furthermore, SA and GABA also showed a positive cross-talk and their foliar spray synergistically enhanced the synthesis of phenolic, flavonoids, putrescinc, and spermidine under Cr stress conditions (Table 2).

**Table 2:** Effect of foliar application of salicylic acid (SA) and gamma aminobutyric acid (GABA) on phenolics, flavonoids, putrescine and spermidine contents barley cultivars grown under chromium stress conditions

Varieties	Treatments	Phenolics (mg g^− 1^ FW)	Flavonoids (mg g^− 1^ FW)	Putrescine (mg g^− 1^ FW)	Spermidine (mg g^− 1^ FW)
Sultan-17	Control	32.80 ± 1.08 fg	19.37 ± 0.76f	1.21 ± 0.051 fg	1.06 ± 0.040ef
	Cr	36.76 ± 1.15e	22.19 ± 1.94def	1.36 ± 0.040def	1.17 ± 0.043de
	Cr + SA	41.36 ± 0.90d	25.43 ± 1.28bcd	1.51 ± 0.075 cd	1.25 ± 0.035bcd
	Cr + GABA	46.28 ± 0.72bc	28.37 ± 0.80bc	1.75 ± 0.046ab	1.36 ± 0.040ab
	Cr + SA + GABA	51.45 ± 0.87a	32.65 ± 1.51a	1.87 ± 0.055a	1.47 ± 0.083a
Haider-93	Control	31.01 ± 0.96 g	18.36 ± 0.34f	1.17 ± 0.062 g	1.01 ± 0.072f
	Cr	35.67 ± 1.24ef	20.35 ± 3.28ef	1.34 ± 0.047ef	1.13 ± 0.077def
	Cr + SA	40.10 ± 0.97d	24.26 ± 0.28cde	1.45 ± 0.041de	1.20 ± 0.034cde
	Cr + GABA	44.83 ± 0.72c	27.23 ± 1.15bc	1.67 ± 0.066bc	1.33 ± 0.035abc
	Cr + SA + GABA	48.37 ± 0.82ab	29.67 ± 0.49ab	1.82 ± 0.065ab	1.42 ± 0.015a

*C*_r_ 40 mg kg^− 1^ soil, *B Yield* Biological yield

Data are presented as mean values ± SE and means sharing different letters are significantly different at *p* < 0.05

### Yield traits

The minimum no of tillers per plant was recorded in Haider-93 (6.87 plant^-1^), and the maximum was recorded in Sultan-17 (7.67 plant^-1^) under Cr stress (Table 3). Salicylic acid and GABA enhanced the number of tillers per plant by 84% and 93% and, and productive tillers per plant by 90% and 91% in Sultan-17 and Haider-93, respectively, under Cr stress, relative to control (Table 3). The Cr stress also caused a sharp decline in spike length, grain yield pot^− 1^,100-grain weight, and biological yield/plant (Table 3). Sultan-17 and Haider-93 showed a reduction of 42% and 46% in spike length under Cr stress with respect to their control (Table 3). Likewise, under Cr stress, grain yield pot^− 1^ significantly decreased by 59% in Sultan-17 and 55% in Haider-93 in comparison to the control (Table 3). Correspondingly, 100 grain weight also reduces by 56% and 61% in Sultan-17 and Haider-93 under Cr stress, while biological yield reduced by 32% and 39% in Sultan-17 and Haider-93, respectively (Table 3). Moreover, the combined exogenous amendments of SA and GABA enhanced spike length (59% and 61%) and 100-grain weight (87% and 92%) and biological yield (38% and 53%) in Sultan-17 and Haider-93, respectively, as compared to Cr stress (Table 3).

**Table 3:** Effect of foliar application of salicylic acid (SA) and gamma aminobutyric acid (GABA) on yield attributes of different barley cultivars grown under chromium stress conditions

Varieties	Treatments	No T/*P*	SL (cm)	No PT/*P*	G Y/*P*	100 GW(g)	B Yield(g)
Sultan-17	Control	11.33 ± 0.334a	7.20 ± 0.258a	10.33 ± 0.058a	23.14 ± 0.026a	5.69 ± 0.072a	30.67 ± 0.351a
	Cr	5.00 ± 0.321de	4.20 ± 0.367 h	4.00 ± 0.032ef	13.87 ± 0.103 h	2.54 ± 0.057gh	21.13 ± 0.575e
	Cr + SA	5.66 ± 0.247de	5.00 ± 0.076f	4.66 ± 0.256def	16.08 ± 0.100f	2.95 ± 0.034ef	23.62 ± 0.329d
	Cr + GABA	6.66 ± 0.054 cd	5.80 ± 0.244d	5.66 ± 0.029de	18.13 ± 0.072d	3.39 ± 0.083d	26.47 ± 0.333c
	Cr + SA + GABA	9.66 ± 0.421ab	6.70 ± 0.179b	7.66 ± 0.334bc	20.62 ± 0.258b	4.77 ± 0.061b	29.31 ± 0.281ab
Haider-93	Control	10.33 ± 0.478ab	7.16 ± 0.643a	9.33 ± 0.126ab	23.12 ± 0.062a	5.67 ± 0.032a	30.41 ± 0.307a
	Cr	4.33 ± 0.298e	3.90 ± 0.041i	3.33 ± 0.531f	12.81 ± 0.182i	2.26 ± 0.027 h	18.57 ± 0.334f
	Cr + SA	5.33 ± 0.065de	4.70 ± 0.437 g	4.33 ± 0.076ef	14.92 ± 0.131 g	2.78 ± 0.055 fg	21.76 ± 0.572de
	Cr + GABA	6.33 ± 0.324 cd	5.60 ± 0.256e	5.33 ± 0.186de	17.06 ± 0.148e	3.11 ± 0.038de	25.62 ± 0.354c
	Cr + SA + GABA	8.00 ± 0.051bc	6.30 ± 0.326c	6.33 ± 0.436 cd	19.36 ± 0.218c	4.35 ± 0.087c	28.50 ± 0.379b

*C*_r_ 40 mg kg^− 1^ soil, *SA* 1 mM, *GABA* 1 mM, *No T/P* No of tillers per plant, *SL* Spike length, *No PT/P* No of productive tillers per plant, *G Y/P* grain yield per pot, *100 GW* 100 grain weight, *B Yield* Biological yield

Data are presented as mean values ± SE and means sharing different letters are significantly different at *p* < 0.05

### Chromium and nutrient accumulation in barley plants

**Fig. 4:**
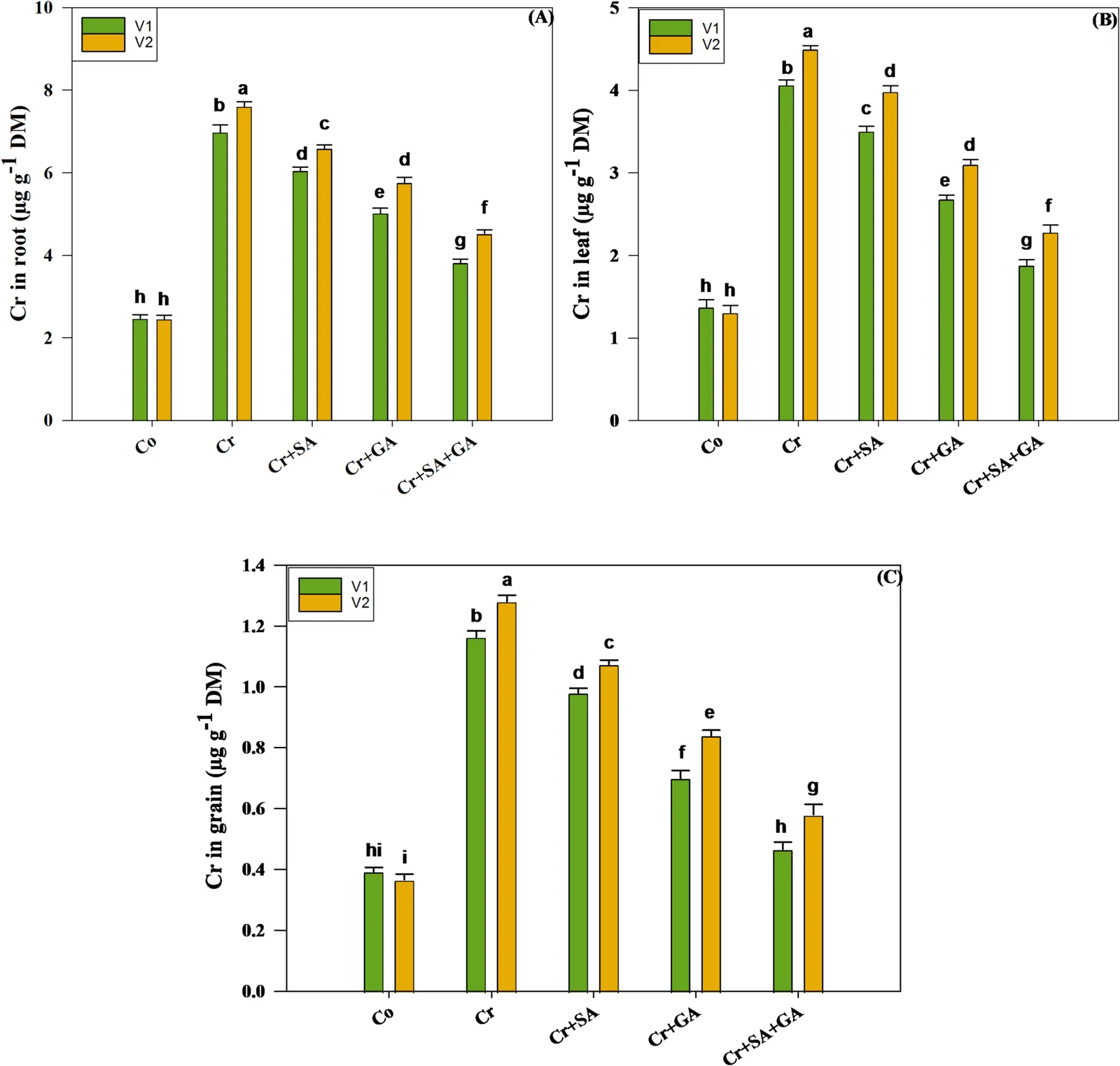
Effect of salicylic acid and GABA on Cr content of different parts root (**a**), leaf (**b**) and grain (**c**) of barley plants subjected to Cr stress (40 mg/kg soil). Each vertical bars represents the means of three replications ± SE. Bars bearing different letters differ significantly at *p* < 0.05. V1: Sultan-17, V2: Haider-93

The maximum Cr concentration in barley root (5.38 µg g^-1^ DW), leaf (3.03 µg g^-1^ DW) and grain (0.83 µg g^-1^ DW) was noted in Haider-93 and minimum Cr concentration in barley root (4.86 µg g^-1^ DW), leaf (2.70 µg g^-1^ DW) and grain (0.74 µg g^-1^ DW) was noted in Sultan-17 (Fig. 4). The combined spray of SA and GABA decreased the Cr concentration in roots (54% and 58%), in leaves (46% and 50%), and in grain (40% and 45%) in Sultan-17 and Haider-93, respectively, under Cr stress as compared to the control (Fig. 4). The results demonstrated that Cr toxicity significantly nutrients concentration in barley cultivars. Notably, Cr stress decreased the N, P, K, Ca, and Mg concentration in Sultan-17 by 78.77%, 87.12%, 47.38%, 53.34%, and 68.63%, respectively (Table 4). Furthermore, Cr stress decreased N, P, K, Ca, and Mg concentration in Haider-93% by 85.49%, 86.30%, 51.40%, 55.96%, and 78.81%, respectively (Table 4). Foliar supplementation of SA and GABA mitigated the adversities of Cr and significantly increased nutrient accumulation in both varieties and co co-applied SA and GABA remained the top-performer (Table 4).

**Table 4:** Effect of foliar application of salicylic acid (SA) and gamma aminobutyric acid (GABA) on nutrient concentration after harvesting of different barley cultivars grown under chromium stress conditions

Varieties	Treatments	Nitrogen	Phosphorus	Potassium	Calcium	Magnesium
	mg kg^− 1^ DW					
Sultan-17	Control	46.75 ± 1.23a	32.99 ± 1.53a	64.48 ± 1.94a	59.07 ± 3.44a	52.90 ± 3.32a
	Cr	26.15 ± 0.37ef	17.63 ± 0.56 fg	43.75 ± 2.32 cd	38.52 ± 1.62d	31.37 ± 1.83de
	Cr + SA	30.62 ± 1.28de	19.16 ± 1.05ef	47.48 ± 4.38 cd	42.33 ± 2.72 cd	35.67 ± 2.41cde
	Cr + GABA	33.31 ± 1.64 cd	22.66 ± 1.35d	59.53 ± 3.87ab	47.32 ± 2.54bc	41.33 ± 2.42bc
	Cr + SA + GABA	41.95 ± 1.79ab	26.09 ± 0.54c	62.45 ± 1.93a	57.51 ± 2.68a	46.54 ± 3.67ab
Haider-93	Control	46.04 ± 1.60a	29.53 ± 1.16b	63.62 ± 2.61a	59.72 ± 2.82a	52.25 ± 2.17a
	Cr	24.82 ± 0.62f	15.85 ± 0.73 g	42.02 ± 3.83d	38.29 ± 1.81d	29.22 ± 1.83e
	Cr + SA	27.97 ± 1.70ef	18.85 ± 1.19efg	44.75 ± 4.02 cd	41.72 ± 2.56 cd	31.47 ± 2.91de
	Cr + GABA	30.95 ± 1.74de	21.15 ± 1.08de	51.62 ± 2.88bc	44.72 ± 3.54 cd	38.55 ± 2.87bcd
	Cr + SA + GABA	37.34 ± 0.81bc	23.68 ± 0.70 cd	61.53 ± 3.65a	54.51 ± 2.70a	44.92 ± 5.02ab

*C*_r_ 40 mg kg^− 1^ soil, *DW* dry weight

Data are presented as mean values ± SE and means sharing different letters are significantly different at *p* < 0.05

### Heatmap and principle components analysis

**Fig. 5:**
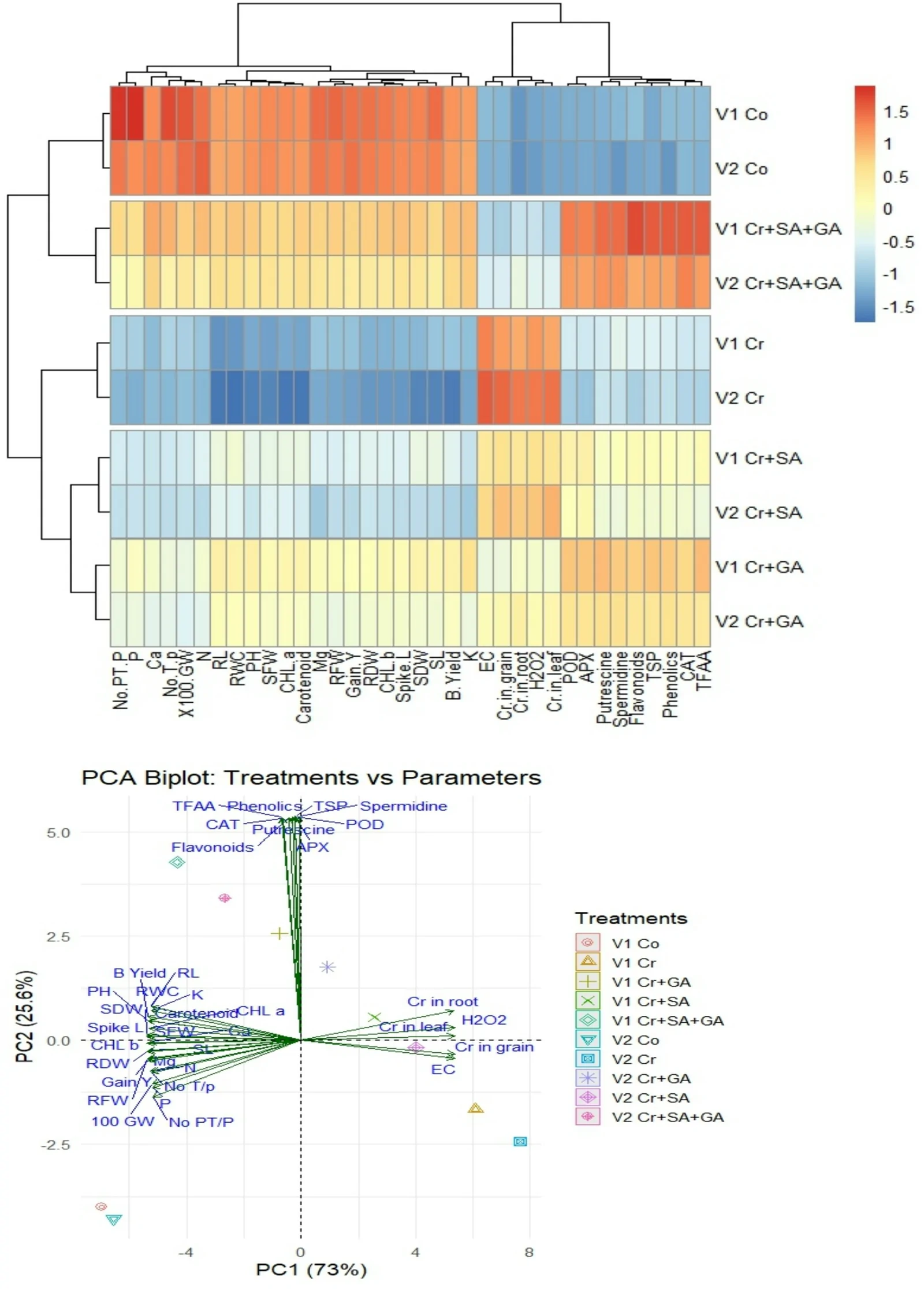
Heat map and PCA between morpho-physiological, biochemical, yield and ionic parameters. PH; Plant Height, SFW; Shoot Fresh Weight, SDW; Shoot Dry Weight, RFW; Root Fresh Weight, RDW; Root Dry Weight, SL; Shoot Length, RL; Root Length CHL a; Chlorophyll a, CHL b; Chlorophyll b, Carotenoids, RWC; Relative Water Content, EL; Electrolyte Leakage, POD; Peroxidase Activity, APX; Ascorbate Peroxidase Activity, CAT; Catalase Activity, TSP; Total Soluble Proteins, TFA; Total Free Amino Acids, H₂O₂; Hydrogen Peroxide, NO T/P; Number of Tillers per Plant, P T/P; Productive Tillers per Plant, 100 GW; Hundred Grain Weight, SL; Spike Length, GY; Grain Yield per Plant, BY; Biological Yield per Plant, Cr L; Chromium in Leaves, Cr G; Chromium in Grains, Cr R; Chromium in Roots

The treatments formed different clusters, with the control plants representing the baseline physiological state. The plants under Cr stress exhibited notable inhibition of morphological parameters and increased expression of stress markers, whereas plants treated with SA and GABA formed independent clusters, suggesting efficient stress mitigation (Fig. 5a). A separate cluster comprising antioxidant enzymes (POD, APX, and CAT) total soluble proteins, and total free amino acids, exhibited higher activity under SA treatment, indicating a strengthened oxidative defense system (Fig. 5). High Cr accumulation in root, shoot, and leaf under Cr toxicity confirmed metal toxicity, whereas lighter shades in growth and yield parameters signified growth decline under Cr stress and recovery with SA and GABA treatments (Fig. 5a). In general, plants treated with SA showed more effective protection than GABA, and the weaker stress responses observed in V1 relative to V2 demonstrated the superior Cr tolerance of Sultan-17 through enhanced anti-oxidative defense and inhibited the uptake of metal (Fig. 5a).

The PCA biplot visualizes the interrelationship between treatments and physiological, biochemical, and morphological traits of barley (Fig. 5a). PC1 and PC2 explained 73 and 25.6% of the total variation, the first two principal component together capturing 98.6% of the overall variability, signifying differentiation among treatments (Fig. 5b). Notably, V1Cr and V2Cr, positioned on the positive axis—highlighting their connection to ionic and oxidative stress conditions (Fig. 5b). Conversely, POD, APX, and CAT, along with TSP and FA, were projected to align along PC2, indicating their greater role in stress mitigation under plants treated with SA and GABA (Fig. 5b). Traits related to growth and yield, including plant height, biomass, RWC, chlorophyll, carotenoids, and grain yield, were associated with the negative axis of PC1, grouping with control and SA-treated plants (Fig. 5b).

## Discussion

The excessive concentration of Cr inhibits plant growth and induces physiochemical changes, necrosis, and chlorosis [45]. Our findings confirmed that Cr inhibited the growth and yield of barley plants, aligning with previous studies [46, 47]. This decrease occurred through a series of mechanisms. Firstly, the increased uptake and accumulation of Cr caused oxidative stress, which damaged the cellular structure, root growth, and elongation. The compromised root growth decreases nutrients and water uptake [45, 48] and disrupts the transportation of essential nutrients. Notably, a deficiency of these nutrients causes a significant decrease in plant growth. Chromium stress also decreased the chlorophyll contents, which limits carbohydrate synthesis and hinders the translocation of photoassimilates to developing grains, decreasing yield [49].

The foliar spray of SA and GABA caused marked enhancement in barley growth (Table 1). The growth promoting effects of SA and GABA was linked with enhanced nutrient absorption, chlorophyll synthesis, and reduced Cr accumulation. The reduction in Cr accumulation was the key mechanism in enhancing the plant growth. This decrease in Cr uptake was possibly linked with the regulation of metal transporter gene expression [50]. The lower Cr accumulation after SA and GABA application decreased the oxidative damages, which preserved the cellular membranes. Consequently, SA and GABA increased the osmotic adjustments, antioxidant defense, and photosynthetic efficiency, leading to better growth and yield [51]. Notably improved photosynthetic efficiency may have increased carbohydrate production, resulting in better growth. In the current study, SA and GABA restored the nutrient homeostasis of barley plants. The increase in P uptake suggested that plants effectively allocated the P to roots for energy demanding processes. Therefore, it increased the growth in response to Cr stress. Moreover, an increase in N uptake favors protein synthesis, while K helps in regulating stomatal movements, reducing water loss, and maintaining CO₂ influx for photosynthesis. Therefore, all these changes contribute to better growth under Cr stress.

The present data revealed a decrease in chlorophyll in response to Cr (Fig. 1), consistent with previous findings reporting that Cr stress induces progressive leaf yellowing and reduces both chlorophyll and carotenoid levels [52]. Chromium inhibits the movement of water, disrupts the mineral uptake (Mg) by the root system, and interferes with enzymatic activities, collectively leading to a reduction in chlorophyll contents [53]. Further, Cr accumulation also triggers oxidative stress, which damages the photosynthetic apparatus, thylakoid membrane structure, and accelerate pigment degradation [53–55]. Exogenous supplementation of SA and GABA notably increased photosynthetic pigments. The observed enhancement in chlorophyll and carotenoid was linked with enhanced antioxidant activities, reduced Cr accumulation, and H_2_O_2_ production [56, 57]. The exogenous supply of SA and GABA significantly enhanced Mg accumulation, which plays a crucial role in chlorophyll synthesis. It is also plausible that SA and GABA alleviated the stomatal limitations by regulating the ion channels (K⁺ and Ca^2⁺^) in guard cells [58]. This regulation may have maintained better gas exchange and contributed to better photosynthetic efficiency in response to Cr stress. Chromium stress lowered RWC and increased EL (Fig. 1), aligning with previous reports [57]. A decline in RWC reflects a turgor loss because of restricted water supply, which is critical for cell enlargement and overall growth. Foliar application of SA and GABA increased RWC and decreased EL caused by Cr (Fig. 1). Foliar spray of SA and GABA improved root growth, thus enhanced water uptake and maintain better RWC. Moreover, they also maintain cellular hydration and membrane integrity [59], thus decreasing the EL production.

Chromium stress caused a significant accumulation of H₂O₂, an important indicator of oxidative damage and lipid peroxidation. These results are aligned with our earlier findings reporting that Cr induces excessive ROS generation, leading to oxidative membrane damage and metabolic imbalance [60, 61]. Notably, lower accumulation in Sultan-17 compared to Haider-93, reflecting its stronger anti-oxidative defense (Fig. 2). Interestingly, barley plants activated the antioxidants in response to Cr, aligning with previous studies [61, 62]. The foliar applied SA and GABA suppressed the accumulation of H₂O₂ through two inter-linked mechanisms: (1) directly by decreasing the Cr uptake and accumulation, (2) increasing the antioxidant activities. Typically, Cr toxicity decreases membrane stability, accompanied by increased MDA production [63]. Our results showed that SA and GABA improved membrane stability by decreasing MDA content (Fig. 2). It is plausible that SA and GABA may have decreased peroxidation of polyunsaturated fatty acids in cell membranes, thereby preserving membrane integrity. Consequently, the production of MDA, a byproduct of lipid peroxidation, is lowered, reflecting the plant’s improved oxidative stress tolerance by SA and GABA [57, 64, 65]. In our study, SA and GABA enhanced the antioxidant activities under Cr stress. Salicylic functions as a regulatory phytohormone that induces ROS-scavenging systems, while GABA acts as a metabolic and signaling molecule that strengthens redox balance by stimulating enzymes involved in the ascorbate–glutathione cycle [66, 67]. Sharma et al. [68] demonstrated that these antioxidant enzymes are pivotal in reducing oxidative stress by scavenging ROS, thereby sustaining redox homeostasis and preventing cellular damage. SA and GABA augmented the activities of key ROS-scavenging enzymes such as SOD, CAT, POD, and APX, thereby strengthening the plant’s antioxidant defense system under Cr stress.

Chromium stress led to a marked reduction in TSP and FAA contents. This decreases was possibly linked with activation of protease, denaturation of proteins, and inhibition of protein synthesis. Notably, the decrease in both traits was more pronounced in Haider-93 compared to Sultan-17 (Fig. 2), indicating that Sultan-17 possess superior stress tolerance traits such as protein system and antioxidants activity. However, foliar supply of SA and GABA enhanced TSP and FAA under Cr. Salicylic acid and GABA stabilize proteins by mitigating oxidative damage, thereby decreasing protein degradation [69, 70]. Secondly, they activate stress-responsive signaling and decrease ROS production, which preserves proteins and enhances their synthesis under stress conditions [71]. The study findings showed that SA and GABA markedly increased the accumulation of secondary metabolites. This aligns with earlier studies reporting that SA and GABA up-regulated the secondary metabolism to counter the stress conditions [59, 72, 73]. The improved synthesis of secondary metabolites scavenge ROS and provides a long-term stress adaptive strategy that enhances plant resilience under stress conditions.

Chromium accumulation was significantly increased in barley plants, and overall Cr concentration followed the order roots > leaves > grains. Chromium translocation within plants is limited compared to cadmium and arsenic; therefore, maximum Cr accumulation was observed in roots [74]. The lower Cr accumulation was observed in leaves and grains, indicating the plants ability to limit Cr movements and protect the vital organs [75, 76]. This differential partitioning is favorable because it localizes the Cr to roots, thereby preserving the shoot and grain functioning. Chromium toxicity significantly decreased the nutrient accumulation in barley plants. The decrease in nutrient uptake was linked with Cr interference with essential nutrients at the root interface, and Cr mediated disrupted ionic balance and competitive binding at transporter sites, thereby decreasing the nutrients adsorption [77, 78]. Interestingly, SA and GABA effectively mitigated the Cr uptake and enhanced nutrient uptake and accumulation in barley plants. SA and GABA promote root lignification [79], which strengthens the cell wall and forms a physical barrier that restricts apoplastic flow and passive metal entry. Co-applied SA and GABA possibly also down-regulated the expression of metal transporters, hence decreasing the Cr accumulation. Furthermore, they may have also regulated the activity of ion channels and down-regulate the genes responsible for metals uptake, thereby decreasing the metal uptake and accumulation. The foliar spray of SA and GABA also enhanced the nutrient uptake. It is well acknowledged that GABA modulates nitrogen metabolism and carbon skeleton availability, therefore improving N assimilation and amino acid synthesis [80]. Additionally, we also observed that the SA and GABA partnership improved the photosynthetic efficiency. This improved photosynthetic efficiency likely supports energy-dependent nutrient uptake mechanisms by maintaining the energy supply.

## Conclusion

Chromium stress significantly suppressed the barley growth, and biomass accumulation by increasing the hydrogen peroxide level, and disrupting photosynthetic pigments, antioxidants activity, and nutrient homeostasis. Among the varieties of barley, Sultan-17 demonstrated higher tolerance to chromium stress, while Haider-93 was the most sensitive cultivar. Foliar treatments with SA and GABA alleviated the adverse effects of Cr and enhanced barley growth and yield. This recovery was attributed to several interconnected mechanisms: (i) reduced Cr accumulation in root and shoot tissues (30–40%), (ii) enhanced synthesis of photosynthetic pigments, osmolytes, and secondary metabolites, (iii) reduction in hydrogen peroxide induced oxidative damages owing to increased antioxidants activities and (iv) maintenance of better nutrient homeostasis in response to Cr stress. Therefore, foliar spray of SA and GABA represents a cost-effective and environmentally friendly strategy to alleviate Cr toxicity and enhance crop productivity. However, this study was conducted in controlled pot conditions; therefore, field validations are required to confirm the efficiency of SA and GABA in different soil and environmental conditions. Furthermore, advanced molecular and omics-based studies are warranted to unravel the precise genetic and biochemical pathways through which SA and GABA synergistically regulate Cr uptake, translocation, detoxification, and stress signaling in plants. 

## Data Availability

The data is contained within the article. Further correspondence can be made to the corresponding author.
